# Perception of Social Support and Disease Acceptance Among Patients Undergoing Cardiac Rehabilitation—Cross-Sectional Study

**DOI:** 10.3390/healthcare13091059

**Published:** 2025-05-04

**Authors:** Patryk Szlacheta, Marika Wlazło, Mateusz Grajek, Magdalena Kłoda-Suchoń, Beata Choromańska-Matera, Antoniya Yanakieva, Ilona Korzonek-Szlacheta

**Affiliations:** 1 Department of Basic Medical Sciences, Faculty of Public Health in Bytom, Medical University of Silesia in Katowice, 41902 Bytom, Poland; 2 Department of Cardiovascular Disease Prevention, Faculty of Public Health in Bytom, Medical University of Silesia in Katowice, 41902 Bytom, Poland; d201206@365.sum.edu.pl (M.W.); ikorzonek@sum.edu.pl (I.K.-S.); 3 Department of Public Health, Faculty of Public Health in Bytom, Medical University of Silesia in Katowice, 41902 Bytom, Poland; mgrajek@sum.edu.pl; 4 Silesian Center for Rehabilitation and Prevention, 43450 Ustroń, Poland; msuchon@scr-ustron.com.pl (M.K.-S.); scr@scr-ustron.com.pl (B.C.-M.); 5 Department of Health Technology Assessment, Faculty of Public Health, Medical University of Sofia, 1000 Sofia, Bulgaria; a.yanakieva@foz.mu-sofia.bg

**Keywords:** cardiac rehabilitation, cardiovascular diseases, disease acceptance, social support

## Abstract

Background: Cardiovascular diseases (CVD), the leading cause of mortality worldwide, require a multidisciplinary approach, with cardiac rehabilitation being a recommended component. The rehabilitation process may be directly influenced by social support, which enhances motivation to cope with the disease and fosters its acceptance. Aims: This study aims to assess the level of social support among patients undergoing cardiac rehabilitation and its impact on disease acceptance. Methods: The study included a sample of 150 patients currently participating in cardiac rehabilitation. Data were collected through direct contact using the validated, anonymous Acceptance of Illness Scale questionnaire, supplemented with a demographic section addressing social support. Results: The majority of respondents reported receiving strong family support (51.3%) and good institutional support (47.3%) during treatment. The mean score for illness acceptance was 29.6 ± 6.9, indicating a high acceptance level observed in most patients (57.3%). The *p*-values for the association between illness acceptance and support from family and institutions were *p* = 0.43 and *p* = 0.82, respectively, suggesting no statistically significant relationship. Conclusions: Patients undergoing cardiac rehabilitation generally experience strong family support, good institutional support, and a high level of disease acceptance. No statistically significant relationship was observed between family or institutional support and the level of disease acceptance.

## 1. Introduction

Acceptance of illness and social support play a pivotal role in the cardiac rehabilitation (CR) process, significantly influencing both program completion and the recovery trajectory of patients with cardiovascular disease (CVD). The present study aims to assess the level of social support among patients undergoing cardiac rehabilitation and to examine its impact on illness acceptance.

Cardiovascular diseases, classified as chronic conditions, remain the leading cause of mortality worldwide. Between 1993 and 2019, the global prevalence of CVD doubled, and in Europe, these diseases account for approximately 49% of all deaths. Current projections estimate that by 2030, CVD-related mortality will reach 23.6 million annually [1,2,3]. Improving patient outcomes requires targeted treatment, encompassing personalized medical care based on individual assessments and predictions of disease progression [4].

Cardiac rehabilitation, recommended as a multidisciplinary intervention, integrates physical, psychological, and social components that have been empirically demonstrated to reduce morbidity and mortality among cardiac patients [5,6,7]. CR programs also include nutritional counseling, smoking cessation strategies, and management of lipid profiles and blood pressure. In European countries, the duration of CR ranges from 8 to 24 weeks, with inpatient programs typically lasting between 3 and 4 weeks [8]. The conventional CR model comprises three phases: inpatient rehabilitation, outpatient physical activity, and long-term unsupervised exercise, preceded by symptom-limited exercise testing. The benefits of CR include enhanced peak oxygen uptake (VO_2_max), improved endurance and endothelial function, reduced risk of depression and anxiety, and overall better quality of life [9].

Social support is a multidimensional construct aimed at mobilizing individuals to cope with illness through the provision of sustained and personalized assistance. Support providers may include family members, friends, co-workers, or neighbors. Research has consistently shown that stronger social support is associated with greater motivation to confront illness and increased hope for recovery [10]. In the context of CR, social support has been linked to improved participation, particularly among older adults, women, and patients with lower socioeconomic status [11]. Additionally, a systematic review by Freak-Poli et al. on the effects of social isolation and support on healthcare utilization and post-cardiovascular event survival suggests that participation in CR may itself enhance perceived social support [12].

Importantly, social support exerts a direct influence on illness acceptance [13], which refers to the patient’s capacity to come to terms with their health status, acknowledge its limitations, and develop effective coping strategies. This process fosters a sense of security and mitigates the psychological burden associated with chronic illness and its treatment [14,15].

## 2. Methods

### 2.1. Study Participants

A cross-sectional study was conducted between November and December 2024 at the Silesian Cardiac Rehabilitation Center in Ustroń, Poland. The study group consisted of 153 patients participating in a cardiac rehabilitation (CR) program, all of whom were in the final phase of their rehabilitation process at the facility. Participants were recruited through purposive sampling among patients currently receiving care at the center. The inclusion criteria were (1) active participation in a CR program at the Silesian Cardiac Rehabilitation Center in Ustroń, (2) being in the final stage of CR, (3) consent to participate in the study, and (4) completion of the questionnaire in accordance with instructions. Exclusion criteria included (1) lack of consent to participate and (2) improperly completed questionnaires. A total of 150 correctly completed questionnaires were included in the final analysis.

### 2.2. Research Tools

The study was conducted through direct contact using the validated Acceptance of Illness Scale (AIS) questionnaire, supplemented with a demographic section that included questions regarding support from family and the institution where treatment was undertaken. The questionnaire was entirely anonymous and did not contain any sensitive information that could identify respondents. The data collection procedure consisted of the following steps:

1. Patients were informed about the voluntary nature of the study by trained healthcare staff.
2. Participants were presented with the aim of the study, the assurance of anonymity and voluntariness, and the fact that refusal to participate would not affect their rehabilitation process.
3. Verbal consent was obtained prior to questionnaire administration.
4. Patients completed the questionnaire in the presence of a trained staff member, who was available to clarify any doubts or difficulties related to question interpretation.
5. Completed questionnaires were collected in a manner that preserved anonymity.

The Acceptance of Illness Scale (AIS) is a research tool adapted into Polish by Juczyński, used to assess disease acceptance among patients suffering from various conditions. The scale consists of eight statements rated on a five-point Likert scale (1—strongly agree; 5—strongly disagree). A low score indicates a lack of disease acceptance, whereas a high score suggests a high level of acceptance. The internal consistency of the test is high, with a Cronbach’s alpha coefficient of 0.82 [16].

- 8–18 points—lack of disease acceptance
- 19–29 points—moderate level of disease acceptance
- ≥30 points—high level of disease acceptance

### 2.3. Statistical Analysis

Statistical analyses were conducted using Statistica 13 and Microsoft Excel. Qualitative variables were presented as the number of observations (*n*) and percentages (%). Associations between categorical variables (e.g., gender, education) and illness acceptance were assessed using Pearson’s chi-square test, while associations with other categorical variables were analyzed using Yates’ corrected chi-square test due to cell sizes below 5. Quantitative data regarding individual items related to illness acceptance and the overall acceptance scores across demographic variables were presented using the mean (M), standard deviation (SD), and median (Me). A significance level of *p* < 0.05 was adopted for all statistical tests.

### 2.4. Ethical Considerations

Prior to the study, an application was submitted to the Bioethics Committee, which confirmed that an ethical review was not required (application no. BNW/NWN/0052/KB/171/24, date 5 August 2024). No sensitive data were collected during the study, and the entire process was conducted in accordance with applicable legal regulations, ensuring full respect for patients and their rights.

## 3. Results

### 3.1. Participant Characteristics

The basic characteristics of the participants are presented in Table 1. The study population consisted of 150 patients undergoing cardiac rehabilitation, including women (*n* = 57, 38%) and men (*n* = 93, 62%). The majority of participants were aged 61–70 years (*n* = 60, 40%). The most frequently reported primary diagnosis was myocardial infarction (*n* = 74, 49.3%), followed by valve replacement surgery (*n* = 34, 22.7%) and heart failure (*n* = 19, 12.7%). These conditions were more prevalent among men (72%) than women (58%). In total, the occurrence of comorbidities was reported (*n* = 79, 52.7%), with diabetes (*n* = 27, 34.2%) and hypertension (*n* = 21, 26.6%) being the most commonly reported (Table 1).

### 3.2. Social Support

A general percentage analysis of responses regarding support from family and the institution where treatment was undertaken, assessed on a scale from “very good” to “very bad” is presented in Table 2.

Among both men and women, “very good” family support was most frequently reported (54.8% and 45.6%, respectively), while “good” institutional support was the most common response (46.2% and 49.1%, respectively). Except for the age group under 30 years, all other groups reported higher ratings for family support compared to institutional support. Participants with higher education most often indicated “very good” support from their family (76.5%), whereas those with primary education predominantly reported “good” support (80%).

### 3.3. Disease Acceptance

This study also analyzed the level of disease acceptance among participants. The mean disease acceptance score was 29.6 ± 6.9. The largest group consisted of individuals with a high level of disease acceptance (86 participants, 57.3%), while 12 participants (8%) did not accept their illness (Table 3).

The results for individual items on the Acceptance of Illness Scale (AIS) are presented in Table 4.

The mean scores for individual statements ranged from 3.31 to 3.97 points. The lowest mean level of disease acceptance, indicating the most frequent agreement among respondents, was observed for the statements “Because of my condition, I can’t do what I like most” (3.31 points) and “I am having trouble adapting to the restrictions imposed by the disease” (3.45 points). Conversely, the highest mean scores (indicating the least agreement) were recorded for the statements “My condition makes me feel incomplete as a person” (3.97 points) and “I think the people around me often feel awkward about my illness” (3.93 points) (Table 4).

An analysis of the relationship between disease acceptance (AIS) and support from family and the treatment institution revealed that respondents with a high level of disease acceptance most frequently reported “very good” family support (61%) and “good” institutional support (57.7%).

A lack of disease acceptance was observed among 9.1% and 10.4% of individuals reporting “very good” support from family and institutions, as well as 8.2% and 5.6% of those indicating “good” support from both sources. No statistically significant relationship was found between family/institutional support and the level of disease acceptance (Table 5).

The level of disease acceptance in relation to demographic characteristics and support from family and institutions is presented in Table 6.

The analysis of disease acceptance results by gender showed that more than half of the surveyed men (58.1%) and women (56.1%) exhibited a high level of disease acceptance. A lack of disease acceptance was reported by 5.3% of men and 12.3% of women, while 36.6% of men and 31.6% of women accepted their disease to a moderate degree.

Regardless of age, individuals most often demonstrated a high level of disease acceptance, observed in 100% of those under the age of 30 and in the 31–40 age group, 66.7% of those aged 41–50, 51.2% of individuals in the 51–60 age range, 60% of those aged 61–70, and 50% of those over 71. Among individuals exhibiting a lack of disease acceptance, 19.5% were in the 51–60 age range, 5% were between 61 and 70, and 3.3% were over 71 years old.

In the studied group, the most frequently reported level of disease acceptance, regardless of education, was high disease acceptance. Among those with a low level of disease acceptance, 1.8% had secondary education, 13.2% had vocational education, and 11.8% had higher education.

A lack of disease acceptance was observed in 14.6% of individuals living in rural areas, 11.4% of those residing in cities with more than 50,000 inhabitants, and 10% of those living in cities with over 200,000 inhabitants. According to this variable, the most frequently reported level of disease acceptance was high, recorded in 63.4% of rural residents, 42.9% of individuals in cities with up to 50,000 residents, 63% of those living in cities with populations between 51,000 and 200,000, and 55% of those residing in cities with over 200,000 inhabitants. Additionally, a statistically significant difference was found between the place of residence and the level of disease acceptance (*p* = 0.01). The lowest average disease acceptance score of 28.6 points was recorded in the group of patients living in cities with over 200,000 residents, while the highest score was found among respondents living in cities with populations between 51,000 and 200,000 (M = 30.9 points).

Individuals who identified as widowed in the marital status question most often exhibited a moderate level of disease acceptance (63.6%), in contrast to those who were married, divorced, or single, who most frequently demonstrated a high level of disease acceptance—59.3%; 55.5%; and 62.5%; respectively. A lack of disease acceptance was found in only 9.7% of married individuals and 5.6% of divorced individuals.

Regardless of the primary diagnosis and type of work undertaken, participants in the study most often displayed a high level of disease acceptance.

Statistically significant differences were observed between employment status and the level of disease acceptance (*p* = 0.02). The lowest average disease acceptance score was recorded in the group of unemployed individuals—20.3 points; while the highest score was obtained by a self-employed individual (35.0 points). A low level of disease acceptance was reported by 66.7% of unemployed individuals, 25% of disability pensioners, 11.5% of working individuals, and 2.5% of retirees. Among the surveyed individuals, a high level of disease acceptance was found in 75% of disability pensioners, 59% of working individuals, 56.2% of retirees, and 33.3% of unemployed individuals.

## 4. Discussion

### 4.1. Social Support

The analysis of the study results revealed that patients undergoing cardiac rehabilitation most frequently reported receiving very good support from their family (51.3%) and good support from the healthcare institution providing treatment (47.3%).

Social support, defined as the provision of emotional and material resources through communication, is a key factor influencing patient engagement in cardiac rehabilitation [17,18]. In a systematic review, McHale et al. highlighted that support from family and friends increased motivation for exercise-based cardiac rehabilitation. The same review also identified healthcare provider support as a determinant of adherence to CR recommendations [19]. Similar findings were reported by Łuczak and Posłuszna-Owcarz, who studied the impact of cardiac rehabilitation on the quality of life in post-myocardial infarction patients. Their analysis showed that the majority of participants (60%) reported receiving support from close family members [20]. High levels of social support were also documented by Blikman et al. in a study conducted at the Krokeide Rehabilitation Center in Norway, where the mean perceived social support score was 5.4 on a 7-point scale [21].

### 4.2. Illness Acceptance

Analysis of the present study data indicated a high level of illness acceptance (scores of 30–40 on the AIS scale) in 57.3% of patients currently undergoing cardiac rehabilitation. A moderate level of illness acceptance (scores of 19–29) was observed in 34.7% of participants, while 8% demonstrated a low level (scores of 8–18). The average illness acceptance score was 29.6 ± 6.9.

The findings did not show a statistically significant relationship between the level of support from the institution providing treatment (*p* = 0.82) or from the family (*p* = 0.43) and illness acceptance. This lack of association may stem from limited variability within the sample, as most participants reported good or very good support from both sources. Additionally, social support may influence illness acceptance indirectly by affecting self-care behaviors. Alizadeh et al., in a study of 170 heart failure patients, identified a significant association between various types of social support and self-care behaviors (*p* < 0.001) [22].

Illness acceptance is a complex psychological construct and a key factor in adherence to medical recommendations. It represents a subjective assessment of one’s health status and facilitates identification of patients’ needs and concerns [23]. Multiple factors influence illness acceptance, including personality traits, health behaviors, and coping strategies [24]. Despite its potential role in rehabilitation outcomes, illness acceptance remains insufficiently explored among patients undergoing cardiac rehabilitation.

The high level of illness acceptance observed in our study group may contribute to patients’ active involvement in the rehabilitation process. However, this relationship may be bidirectional. Sanaie et al., in a qualitative study on patient engagement in CR, suggested a two-way relationship between CR and illness acceptance. Their findings showed that greater illness acceptance can lead to increased participation in rehabilitation, while engagement in CR may, in turn, enhance illness acceptance and treatment adherence. Due to the lack of pre-rehabilitation data and the cross-sectional design of our study, we cannot determine the direction of this relationship [25].

Although few studies specifically focus on illness acceptance in the context of CR, some have explored this issue among general cardiac patient populations [13,23,26,27,28,29,30]. Dugunchi et al. assessed treatment adherence, illness acceptance, and illness perception in 280 patients with coronary artery disease. Using the AIS scale, they reported a mean illness acceptance score of 16.98 ± 4.75, indicating a relatively low level. Contrasting with our findings, where the mean was significantly higher. Additionally, Dugunchi et al. identified a significant association between marital status and illness acceptance (OR = 0.068 and 0.148, respectively), a finding not supported by our data (*p* = 0.29) [26].

Obiegło et al. studied illness acceptance in patients with chronic heart failure, reporting results consistent with ours: 40% of participants had a high level of illness acceptance, and 36% had a moderate level [11]. Similarly, Jankowska-Polańska, in a study of 105 hospitalized hypertensive patients, found that 41% exhibited high and 40% moderate levels of illness acceptance [27].

In contrast, Baczewska et al., in a study of 154 hypertensive patients, found that most (68.8%) had moderate illness acceptance, while 13.0% had low and 18.2% had high acceptance. Their study also noted that the statement “I think people around me are often embarrassed because of my illness” was among the most disagreed with (mean score: 3.4) [28]. This was echoed by Białek and Dadowski, who also reported strong disagreement with this statement (mean score: 4.36) [29]. Our results are consistent, as this statement also received one of the highest mean scores in our sample (3.93).

Pluta et al. explored illness acceptance among patients with hypertension in a sample of 200 individuals at two healthcare centers in Bydgoszcz. They found high and moderate illness acceptance levels in 62% and 33% of participants, respectively. They also observed gender differences: moderate acceptance was found in 30% of men and 35.7% of women, suggesting that women may face greater difficulty accepting their illness. This contrasts with our findings, where moderate acceptance was more prevalent among men than women (36.6% vs. 31.6%). Furthermore, low illness acceptance was reported by only 1.2% of men, compared to 7.8% of women. Good illness acceptance was reported by 69.4% of men and 56.5% of women, indicating higher acceptance among men. These results align with our study, where women more often reported lower illness acceptance (12.3% vs. 5.3%), while men more frequently reported high illness acceptance (58.1% vs. 56.1%) [23]. However, the relationship between gender and illness acceptance was not statistically significant (*p* = 0.30), highlighting the need for further research in this area.

Our study did not confirm a statistically significant association between family or institutional support and illness acceptance. This contrasts with findings from other authors, as previously discussed. For example, Karataş and Bostanoğlu observed a moderate correlation between perceived social support and psychosocial adaptation among patients with ischemic heart disease. They noted that increased support was associated with better psychosocial adjustment (r = −0.523, *p* < 0.05) [30].

The aim of our study was to assess the level of social support among patients undergoing cardiac rehabilitation and its relationship to illness acceptance. The findings suggest that most participants experienced high or moderate illness acceptance and good or very good social support from both family and the institution providing care. However, no statistically significant relationship between support and illness acceptance was found, possibly due to the homogeneity of the sample. These results emphasize the need for further investigation into this complex issue.

## 5. Strengths and Limitations

The described study has both strengths and limitations. The use of a validated tool for assessing disease acceptance (AIS) and the inclusion of patients actively participating in cardiac rehabilitation—an important yet underexplored clinical group—constitute a major strength of this research. Nonetheless, caution must be exercised when interpreting the findings, as the study sample was not representative of the broader population. Additionally, the study was conducted at a single center, limiting the generalizability of the results. The present study did not include information regarding the duration of the disease or the nature of its onset (sudden or gradual), both of which may directly affect the level of disease acceptance among the participants. Moreover, the study was conducted among patients in the final period of rehabilitation, which could directly translate into the level of disease acceptance.

Future research should involve larger and more diverse patient cohorts, encompassing participants from multiple institutions. Such an approach would enhance the external validity of the findings and facilitate a more comprehensive understanding of the studied phenomenon. In the future, it would be advisable to conduct studies that provide a broader view of the course of the disease, allowing for the identification of additional factors potentially influencing the level of disease acceptance.

## 6. Conclusions

The vast majority of patients undergoing cardiac rehabilitation experience strong social support from both their families and healthcare institutions, with support levels generally rated as good or very good. Our findings indicate a high level of disease acceptance among survey participants. Additionally, the most frequently reported challenges related to disease acceptance included difficulties in adapting to the limitations imposed by the illness and restrictions on engaging in preferred activities due to health status. Disease acceptance was significantly associated with place of residence and employment status. The lowest mean disease acceptance score was observed among patients residing in cities with populations exceeding 200,000, while the highest was recorded among respondents living in cities with populations between 51,000 and 200,000. Unemployed individuals exhibited the lowest mean disease acceptance score, whereas self-employed participants demonstrated the highest score. No statistically significant association was found between family or institutional support and disease acceptance. The lack of a statistically significant association may result from the homogeneity of the study sample. In order to gain a more accurate understanding of the relationship between social support and illness acceptance among patients undergoing cardiac rehabilitation, it is recommended to conduct broader cross-sectional studies involving a more diverse sample.

## Figures and Tables

**Table 1:** Characteristics of the study population.

Category	Value	Group Size *n* = 150 (%)
Gender	Male	93 (62%)
	Female	57 (38%)
Age	≤30 years	1 (0.7%)
	31–40 years	3 (2%)
	41–50 years	15 (10%)
	51–60 years	41 (27.3%)
	61–70 years	60 (40%)
	≥71 years	30 (20%)
Education	Primary	6 (4%)
	Secondary	57 (38%)
	Professional	53 (35.3%)
	Higher	34 (22.7%)
Place of Residence	Village	41 (27.3%)
	Town (<50,000 inhabitants)	35 (23.3%)
	City (51,000–200,000 inhabitants)	54 (36%)
	City (>200,000 inhabitants)	20 (13.3%)
Marital Status	Married	113 (75.3%)
	Divorced	18 (12%)
	Widowed	11 (7.3%)
	Single	8 (5.3%)
Employment Status	Retired	80 (53.3%)
	Employed	61 (40.7%)
	Disability pensioner	4 (2.7%)
	Unemployed	3 (2%)
	Other	2 (1.3%)
Type of Work	Physical labor	60 (40%)
	Mental work	38 (25.3%)
	Not applicable	44 (29.3%)
	Other	8 (5.3%)

**Table 2:** Support from family and institutions.

Support Type	Level	*n*	%
Family Support	Very good	77	51.3
	Good	61	40.7
	Satisfactory	12	8
	Bad	0	0
	Very bad	0	0
Institutional Support	Very good	48	32
	Good	71	47.3
	Satisfactory	31	20.7
	Bad	0	0
	Very bad	0	0

**Table 3:** Distribution of the study group by disease acceptance level.

Acceptance Level	Score Range	*n*	%
Lack of acceptance	8–18 points	12	8
Moderate acceptance	19–29 points	52	34.7
High acceptance	≥30 points	86	57.3

**Table 4:** Statement results for disease acceptance.

Statement	M *	SD **	Me ***
I am having trouble adapting to the restrictions imposed by the disease	3.45	1.23	4.0
2. Because of my condition, I can’t do what I like most	3.31	1.22	4.0
3. The disease sometimes makes me feel unnecessary	3.85	1.27	4.0
4. Health problems make me more dependent on others than I would like to be	3.49	1.30	4.0
5. The disease makes me a burden to my family and friends	3.91	1.29	4.0
6. My condition makes me feel incomplete as a person	3.97	1.25	4.0
7. I will never be self-sufficient in the way I would like to be	3.71	1.21	4.0
8. I think the people around me often feel awkward about my illness	3.93	1.22	4.0

* M—Mean. ** SD—Standard deviation. *** Me—Median.

**Table 5:** Disease acceptance level considering the direction of support.

Support Type	Support Level	Acceptance Level						
		Lack of Acceptance		Moderate Acceptance		High Acceptance		*p*-Value
		*n*	%	*n*	%	*n*	%	
Family Support	Very good	7	9.1	23	29.9	47	61	0.43
	Good	5	8.2	23	37.7	33	54.1	
	Satisfactory	0	0	6	50	6	50	
	Bad	0	0	0	0	0	0	
	Very bad	0	0	0	0	0	0	
Institutional Support	Very good	5	10.4	17	35.4	26	54.2	0.82
	Good	4	5.6	26	36.6	41	57.7	
	Satisfactory	3	9.7	9	29	19	61.3	
	Bad	0	0	0	0	0	0	
	Very bad	0	0	0	0	0	0	

**Table 6:** Disease acceptance in relation to demographic characteristics and primary diagnosis.

**Category**	Support Level	Acceptance Level							
		Lack of Acceptance		Moderate Acceptance		High Acceptance		M—Mean Score	*p*-Value
		*n*	%	*n*	%	*n*	%		
Gender	Male	5	5.3	34	36.6	54	58.1	30.2	0.30
	Female	7	12.3	18	31.6	32	56.1	28.7	
Age	≤30 years	0	0	0	0	1	100	32.0	0.10
	31–40 years	0	0	0	0	3	100	30.7	
	41–50 years	0	0	5	33.3	10	66.7	30.4	
	51–60 years	8	19.5	12	29.3	21	51.2	28.7	
	61–71 years	3	5	21	35	36	60	29.9	
	≥71 years	1	3.3	14	46.7	15	50	29.4	
Education	Primary	0	0	3	50	3	50	30.0	0.19
	Secondary	1	1.8	22	38.6	34	59.6	30.7	
	Professional	7	13.2	17	32.1	29	54.7	28.6	
	Higher	4	11.8	10	29.4	20	58.8	29.3	
Place of Residence	Village	6	14.6	9	22	26	63.4	28.9	0.01
	Town (<50,000 inhabitants)	4	11.4	16	45.7	15	42.9	28.9	
	City (51,000–200,000 inhabitants)	0	0	20	37	34	63	30.9	
	City (>200,000 inhabitants)	2	10	7	35	11	55	28.6	
Marital Status	Married	11	9.7	35	31	67	59.3	29.6	0.29
	Divorced	1	5.6	7	38.9	10	55.5	30.3	
	Widowed	0	0	7	63.6	4	36.4	28.5	
	Single	0	0	3	37.5	5	62.5	29.0	
Employment Status	Retired	2	2.5	33	41.3	45	56.2	30.4	0.02
	Employed	7	11.5	18	29.5	36	59	28.9	
	Disability pensioner	1	25	0	0	3	75	30.8	
	Unemployed	2	66.7	0	0	1	33.3	20.3	
	Other	0	0	1	50	1	50	35.0	
Type of Work	Physical labor	7	11.7	21	35	32	53.3	28.6	0.67
	Mental work	2	5.3	14	36.8	22	57.9	30.4	
	Not applicable	3	6.8	13	29.5	28	63.6	30.3	
	Other	0	0	4	50	4	50	30.7	
Primary diagnosis	Myocardial infarction	6	8.1	27	36.5	41	55.4	29.1	0.49
	Exchange valve	3	8.8	13	38.2	18	52.9	28.9	
	Heart failure	2	10.5	4	21.1	13	68.4	31.8	
	Other	1	4.3	8	34.8	14	60.9	31.4	

## Data Availability

The original contributions presented in this study are included in the article. Further inquiries can be directed to the corresponding author(s).
